# FGF2-FGFR1 pathway activation together with thymidylate synthase upregulation is induced in pemetrexed-resistant lung cancer cells

**DOI:** 10.18632/oncotarget.26622

**Published:** 2019-02-05

**Authors:** Kentaro Miura, Takaaki Oba, Kazutoshi Hamanaka, Ken-ichi Ito

**Affiliations:** ^1^ Division of Breast, Endocrine and Respiratory Surgery, Department of Surgery (II), Shinshu University School of Medicine, Matsumoto, Japan

**Keywords:** drug resistance, FGFR pathway, lung cancer, pemetrexed, epithelial-mesenchymal transition

## Abstract

Pemetrexed (MTA) is a folate antimetabolite used for treating non-small cell lung cancer. To elucidate the mechanisms of pemetrexed resistance in lung cancer, we established pemetrexed-resistant sublines in PC9 (mutant *EGFR*) and H1993 (wild-type *EGFR*) lung adenocarcinoma cell lines (PC9-MTA, H1993-MTA). Gene expression profile comparison by microarray analyses revealed enhanced fibroblast growth factor 2 (FGF2) and FGF receptor 1 (FGFR1) expression, confirmed by Western blotting, enzyme-linked immunosorbent assay, and reverse transcription-polymerase chain reaction. ERK phosphorylation was increased in PC9-MTA but decreased in H1993-MTA along with decreased downstream signaling molecule phosphorylation. Cellular morphological change from epithelial to spindle-shape together with increased mesenchymal marker protein expression was observed in H1993-MTA. SiRNA-mediated FGF2 knockdown partially restored pemetrexed sensitivity in both lines, whereas anti-FGFR1 inhibitor PD173074 restored pemetrexed sensitivity in PC9-MTA. FGF2 or FGFR1 inhibition decreased pERK levels in PC9-MTA but increased pEGFR levels together with downstream signaling molecule activation and reversed epithelial-mesenchymal transition marker protein expression in H1993-MTA. Although thymidylate synthase strongly facilitates the development of pemetrexed resistance, our results reveal involvement of the FGF2-FGFR1 pathway in pemetrexed resistance in lung cancer cells and suggest that cellular function alterations induced by FGF2-FGFR1 pathway activation depend on the innate feature of cancer cells.

## INTRODUCTION

Primary lung cancer constitutes the most frequent cause of cancer-related deaths worldwide and is the leading cause of cancer-related deaths among men and the second leading cause among women in Japan. However, although the survival of patients with lung cancer has gradually improved, patient prognosis remains far from favorable.

Pemetrexed is a thymidylate synthase (TS) inhibitor that has been widely used for non-small cell lung cancer (NSCLC) excluding squamous cell lung cancer to suppress DNA synthesis by decreasing turnover of dUMP to dTMP [1, 2, 3]. It also inhibits dihydrofolate reductase and glycinamide ribonucleotide formyltransferase. Recently, the association between TS elevation and pemetrexed resistance has been reported *in vitro* [4, 5, 6]. Moreover, in a clinical setting, Liu et al. reported that the increasing level of TS likely served as an independent risk factor of potential resistance against pemetrexed in NSCLC by meta-analysis [7]. Thus, it is highly possible that the overexpression of TS plays an important role in pemetrexed resistance.

Among other mechanisms involved in pemetrexed resistance, Uemura et al. demonstrated that ATP-binding cassette-transporter 11 (ABCC11) may represent a biomarker for pemetrexed treatment in adenocarcinomas and that a single-nucleotide polymorphism in the *ABCC11* gene constitutes an important determinant of pemetrexed sensitivity [8]. In addition, Chiu et al. recently reported that the acquisition of pemetrexed resistance enhances the epithelial-mesenchymal transition (EMT) of lung cancer cells accompanied with upregulation of ZEB1 and fibronectin together with downregulation of E-cadherin and extracellular signal-regulated kinase (ERK) 1/2 *in vivo* and *in vitro* [5].

The enhanced expression of fibroblast growth factors (FGFs), which constitute a large family of growth factors that play a variety of roles in cellular differentiation, cell growth, and embryogenesis [9, 10, 11], as well as that of FGF receptors (FGFRs) has also been reported in NSCLC cell lines [12, 13, 14]. In particular, FGF2 functions as a potent angiogenic factor that acts as both a mitogen and an activator of epithelial cell migration [15]. Moreover, recent studies have revealed that the FGF2-FGFR1 autocrine pathway is involved in the acquired resistance to EGFR-tyrosine kinase inhibitors (TKIs) such as gefitinib and afatinib in *EGFR* mutation positive NCSLC cell lines [14, 16]. However, whether the FGF2-FGFR1 pathway is involved in the mechanism of acquisition of pemetrexed resistance has not yet been elucidated.

To elucidate the mechanisms underlying the development of pemetrexed resistance in NSCLC, we established two pemetrexed-resistant sublines in two lung cancer cell lines, one carrying an *EGFR* mutation and the other retaining wild-type *EGFR*. In the present study, we demonstrated for the first time that the upregulation of the FGF2-FGFR1 pathway could confer pemetrexed resistance in lung cancer cells regardless of *EGFR* status.

## RESULTS

### Establishment of pemetrexed-resistant lung cancer cell lines

**Figure 1:**
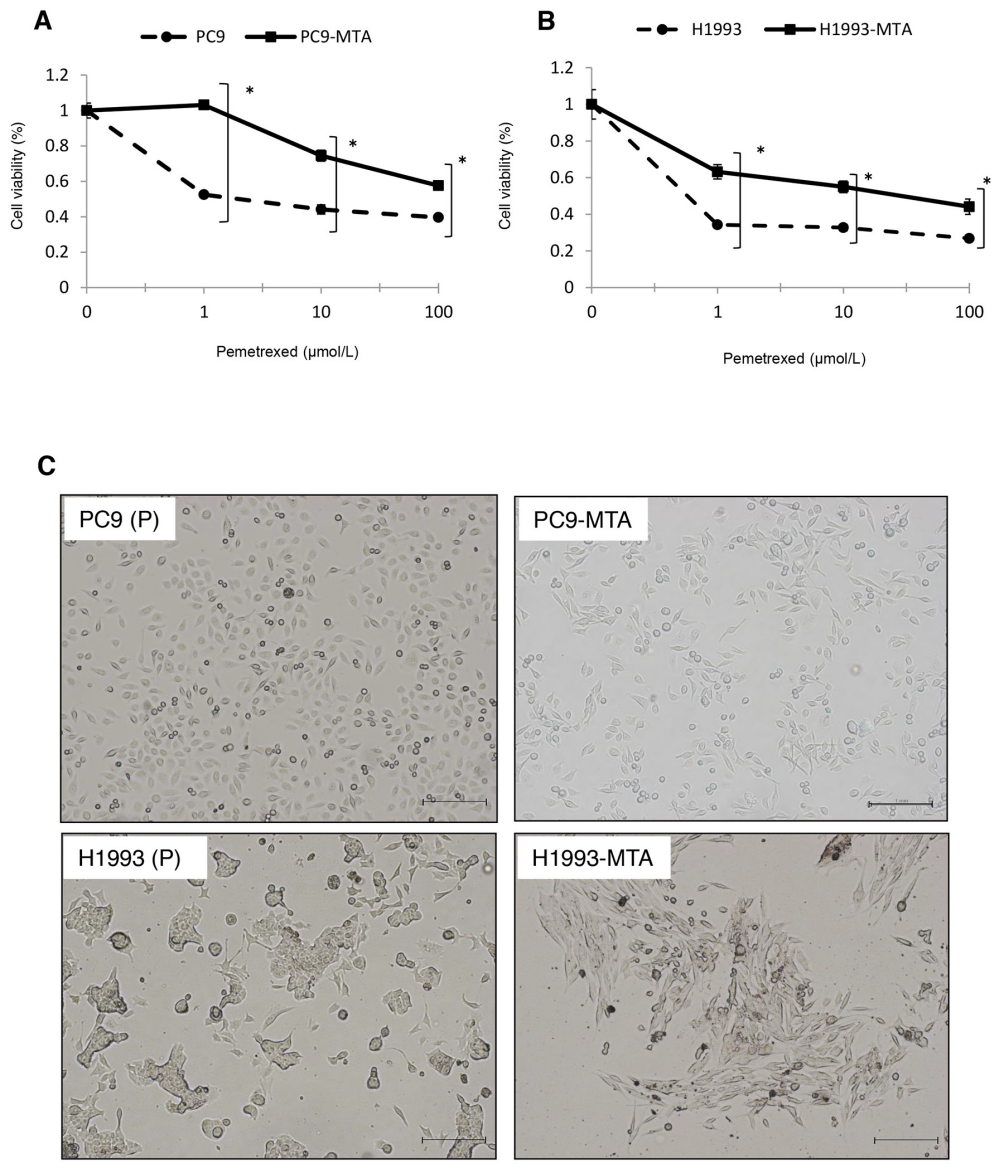

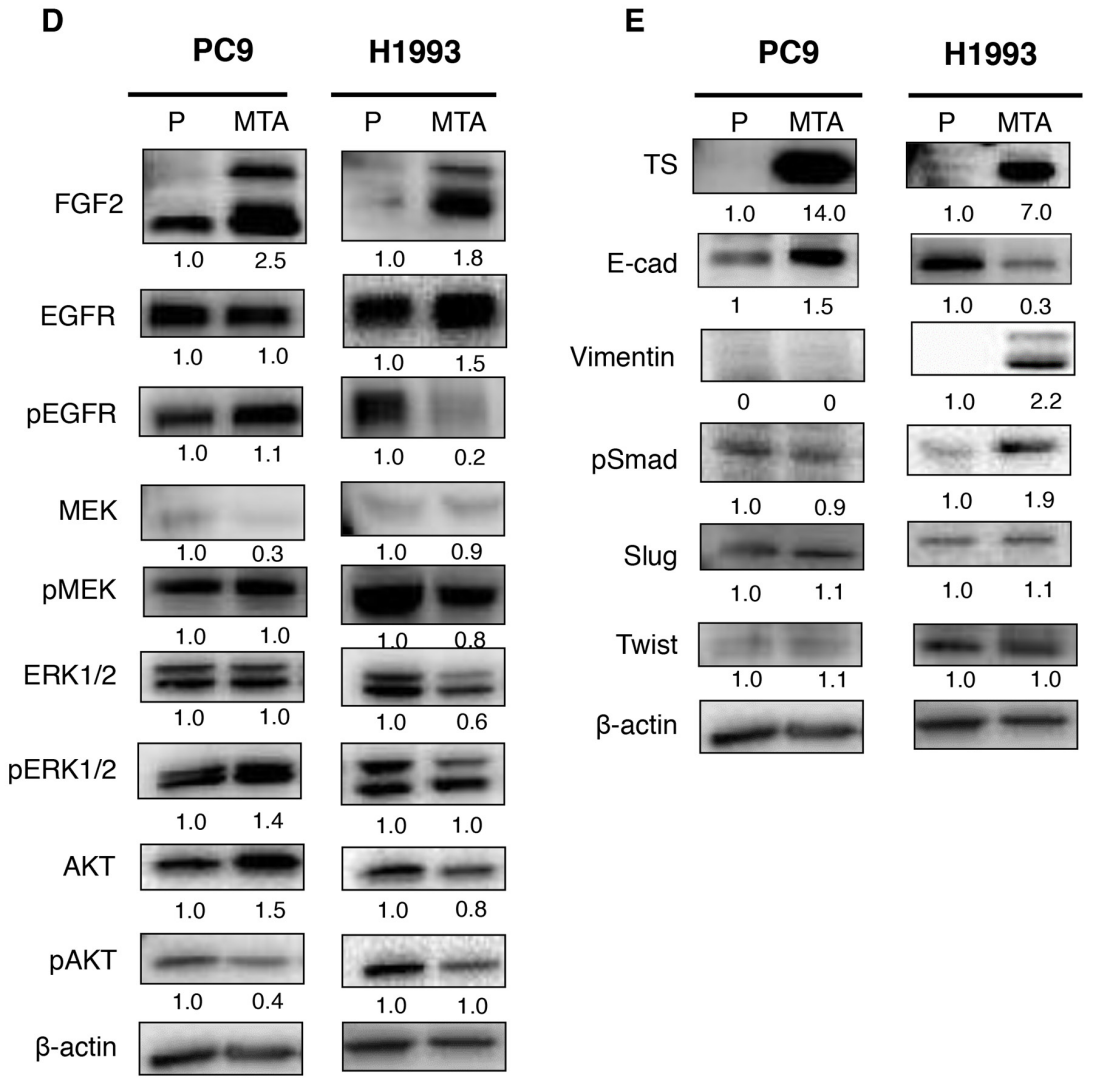

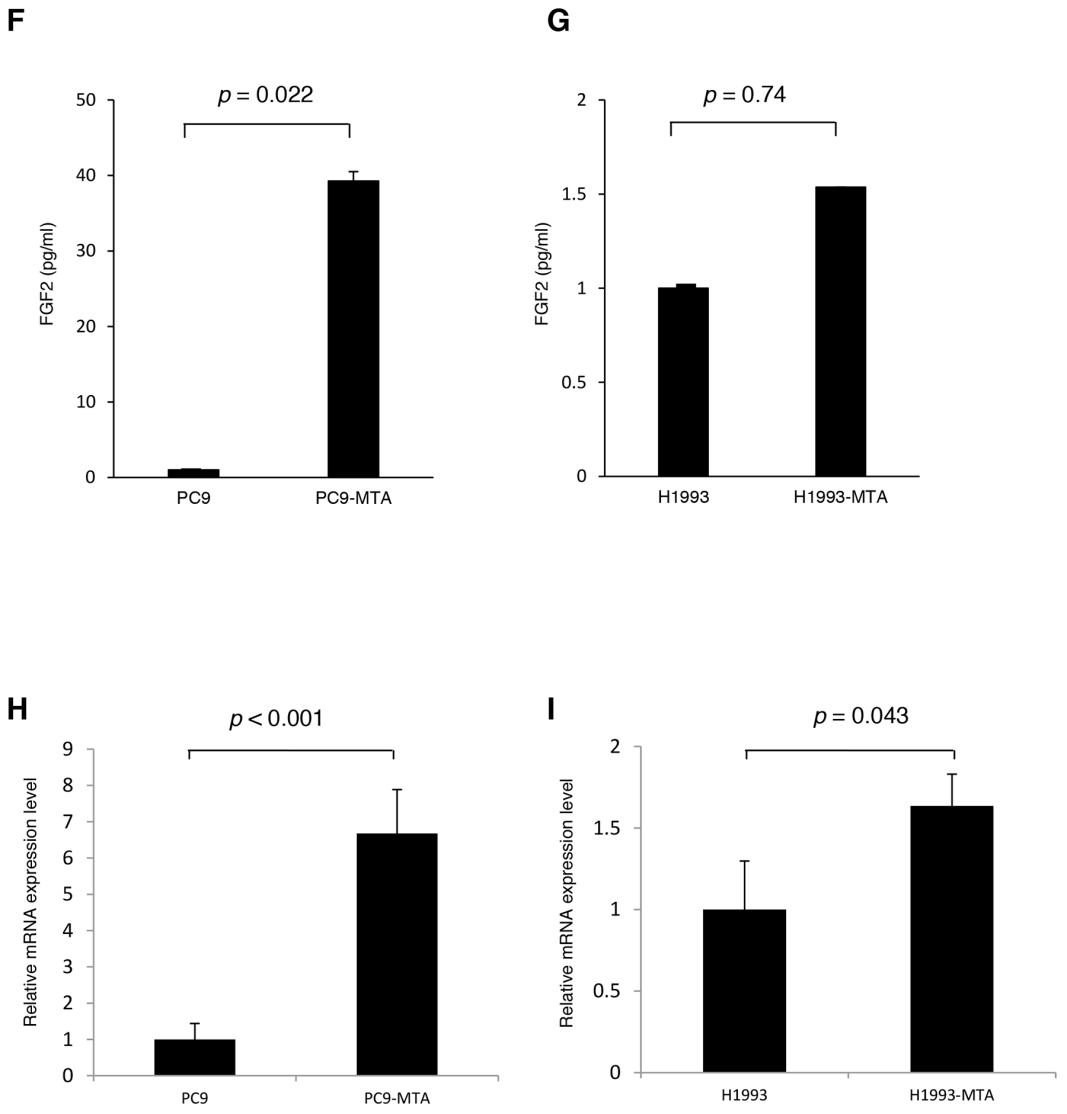
Characteristics of pemetrexed-resistant lung cancer sublines and their parental cells Sensitivity to pemetrexed in pemetrexed-resistant lung cancer sublines and their parental cells. **(A, B)** Pemetrexed-resistant lung cancer cell lines were obtained by culturing PC9 and H1993 cells with stepwise-increasing doses of pemetrexed for over 6 months. Sensitivity to pemetrexed was determined by using WST assays. Each cell line with “P” indicates a parental cell line, and “-MTA” indicates an established pemetrexed-resistant subline. Closed circles (●) indicate parental cells, whereas closed squares (■) indicate pemetrexed-resistant cells. The error bars represent the standard error of the value obtained in the experiments performed in triplicate.Morphological findings of pemetrexed-resistant lung cancer sublines and their parental cells. **(C)** Representative pictures of the morphological findings of the parental PC9 cells (PC9-P), PC9-MTA cells, parental H1993 cells (H1993-P), and H1993-MTA cells. Scale bars = 500 μm. Comparison of signaling pathway molecules and EMT marker proteins between parental and pemetrexed-resistant lung cancer cells. **(D)** Western blot analyses of the expression of total or phosphorylated forms (pEGFR, pMEK, pERK, and pAKT) of signaling molecules in the parental PC9 cells (PC9-P), PC9-MTA cells, parental H1993 cells (H1993-P), and H1993-MTA cells. β-actin was used as a loading control.The experiments were repeated independently at least three times, and one representative blot is provided in the figures. The quantitative numbers of relative expression levels corrected by β-actin are demonstrated below the picture of the blots. The phosphorylated proteins were normalized to their total amounts. **(E)** Western blot analyses of the expression of TS and EMT marker proteins in PC9-P, PC9-MTA, H1993-P, and H1993-MTA cells. **(F, G)** Comparison of FGF2 protein expression levels in serum-free conditioned media measured by ELISA between PC and PC9-MTA cells **(F)** and between H1993 and H1993-MTA cells **(G). (H, I)** *FGFR1* expression quantitated by real-time RT-PCR in PC9 and PC9-MTA cells **(H)** and in H1993 and H1993-MTA cells **(I)**. The error bars in each graph represent the standard error of the value obtained in the experiments performed in triplicate.

Pemetrexed-resistant lung cancer cell lines were obtained by culturing PC9 [*EGFR* exon 19 deletion (delE746-A750)] and H1993 [*EGFR* wild-type] cells with stepwise increases in the pemetrexed concentration for over six months; the pemetrexed-resistant sublines were designated as PC9-MTA and H1993-MTA, respectively. The relative pemetrexed resistance of PC9-MTA and H1993-MTA compared to the corresponding parental cell line was determined using a tetrazolium salt-based proliferation (WST) assay (Figure 1A, 1B). The IC_50_ for the parental PC9 and H1993 lines were 1.30 ± 0.26 and 0.05 ± 0.02 μM, whereas thosefor the PC9-MTA and H1993 were > 100 and 7.30 ± 0.03 μM, respectively (Table 1). Thus, PC9-MTA and H1993-MTA exhibited over 77-fold and 146-fold greater pemetrexed resistance than that of their respective parental cell lines.

**Table 1:** IC_50_ for pemetrexed (MTA) in the parental and pemetrexed-resistant lung cancer cell lines

Cell line	PC9	PC9-MTA	H1993	H1993-MTA
IC_50_ (μM)^a^ (mean ± SD)	1.30 ± 0.26	> 100	0.05 ± 0.02	7.30 ± 0.03
Relative resistance ratio^b^		> 77		146

^a^IC_50_: half-maximal inhibitory concentration

^b^Relative resistance ratio = IC_50_ of pemetrexed-resistant cells/IC_50_ of parental cells

### Morphological observation of the parental and pemetrexed-resistant lung cancer cells

Figure 1C shows the morphological observations of PC9 and H1993 cells. No obvious morphological change was observed between the parental PC9 and PC9-MTA cells. Conversely, H1993 cells changed from a classical epithelial morphology to a spindle-shaped morphology after the cells acquired resistance to pemetrexed.

### Pemetrexed-resistant sublines showed increased expression of FGF2, FGFR1, and TS

In the present study, we compared the gene expression profile between PC9 and PC9-MTA cells, and between H1993 and H1993-MTA cells by microarray analysis. We found that the expression of *FGF2* in PC9-MTA and H1993-MTA cells increased 21.8-fold and 28.4-fold, respectively, compared to that in the parental cell lines (data not shown). Based on this result, we examined the expression of FGF2 by Western blotting and confirmed that this was drastically increased in both pemetrexed-resistant cell lines (Figure 1D, Supplementary Figure 1). Furthermore, we measured the protein expression levels of FGF2 in serum-free conditioned media in PC9, PC9-MTA, H1993, and H1993-MTA cells (Figure 1F, 1G). PC9-MTA cells produced around 30-fold higher levels of FGF2 than the parental PC9 cells, and H1993-MTA cells produced around 1.5-fold higher level of FGF2 than the parental H1993 cells, indicating that functional FGF2 protein was secreted by these pemetrexed-resistant cell lines.

In addition, the results of microarray analysis demonstrated that *FGFR1* expression was increased 2.2-fold in PC9-MTA and 3.8-fold in H1993-MTA cells compared with that in their parental cell lines. Significantly increased *FGFR1* expression was also confirmed through quantitation by real-time reverse transcription-polymerase chain reaction (RT-PCR) between the pemetrexed-resistant and parental sublines (*p* < 0.001, *p* = 0.043, respectively) (Figure 1H, 1I). These results indicated that the FGF2-FGFR1 pathway was upregulated upon PC9 and H1993 cell acquisition of resistance to pemetrexed. Furthermore, the expression of TS was highly enhanced in the pemetrexed-resistant sublines established in the present study, which was consistent with the findings demonstrated in previous studies (Figure 1E, Supplementary Figure 2).

### Expression of proteins related to signal transduction pathways

Next, we examined whether the expression or phosphorylation of the downstream signaling molecules of the FGF2-FGFR1 pathway including the mitogen-activated protein/extracellular signal–regulated kinase (MAPK/ERK) and phosphoinositide 3-kinase (PI3K)-AKT pathways were altered along with the development of pemetrexed-resistance by Western blotting (Figure 1D). A marked increase of FGF2 expression was observed upon acquisition of pemetrexed resistance in both PC9 and H1993 cells; however, the phosphorylation of proteins in the signaling pathway was different between the two cell lines. In PC9-MTA cells, which carry an *EGFR* mutation, the phosphorylation of EGFR was unchanged, whereas this was markedly reduced in H1993-MTA cells. Furthermore, the phosphorylation of ERK1/2 was elevated in PC9-MTA cells. In contrast, the phosphorylation of ERK, MEK, as well as AKT was decreased in H1993-MTA cells. These results suggest that reduced phosphorylation of EGFR might induce the inhibition of downstream signaling molecules in both MAPK/ERK and PI3K-AKT pathways in H1993-MTA cells, and that the signaling pathway alteration caused by the increase of FGF2 might differ according to the molecular features of each lung cancer cell line.

### Expression of EMT molecular markers in pemetrexed-resistant lung cancer cells

As the morphological change from a classical epithelial shape to a mesenchymal spindle-shape was observed in the pemetrexed-resistant cells, the expression of EMT molecular marker proteins was examined by Western blotting (Figure 1E). In H1993 cells, E-cadherin was decreased whereas the phosphorylation of Smad and the expression of vimentin were increased when the cells acquired resistance to pemetrexed. These results were consistent with the acquisition of mesenchymal morphology by the H1993-MTA cells. In contrast, the expression of E-cadherin was increased in the PC9-MTA cells whereas no alteration of pSmad, Slug, or vimentin expression was observed. Thus, our data indicated that the induction of phenotype switch during the development of resistance to pemetrexed might depend on the innate character of the lung cancer cells.

### Restoration of pemetrexed sensitivity by FGF2 or TS knockdown in pemetrexed-resistant lung cancer cells

**Figure 2:**
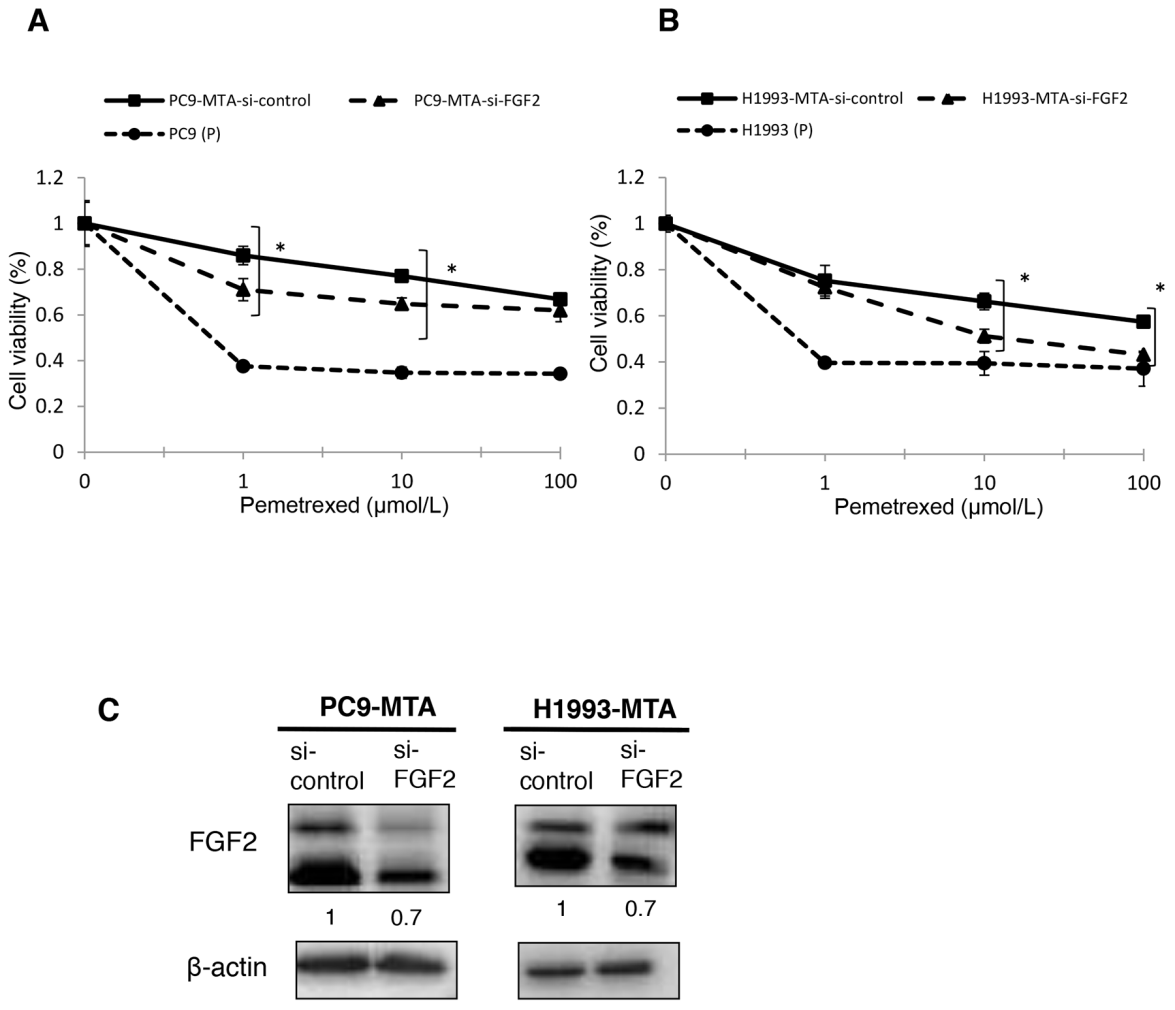
Effects of FGF2 knockdown in pemetrexed-resistant lung cancer cells The expression of FGF2 in PC9-MTA and H1993-MTA cells was inhibited by siRNA, and the sensitivity to pemetrexed was tested using WST assays. **(A)** Sensitivity to pemetrexed was measured in PC9-MTA cells transfected with siRNA targeting control (si-control) or FGF2 (si-FGF2) and the parental PC9 cells (P). **(B)** Sensitivity to pemetrexed was measured in H1993-MTA cells transfected with siRNA (si-control or si-FGF2) and the parental H1993 cells (P). The error bars in each graph represent the standard error of the value obtained in the experiments performed in triplicate. **(C)** FGF2 expression as determined by the Western blot analyses in PC9-MTA cells and H1993-MTA cells transfected with si-FGF2 or si-control.

**Figure 3:**
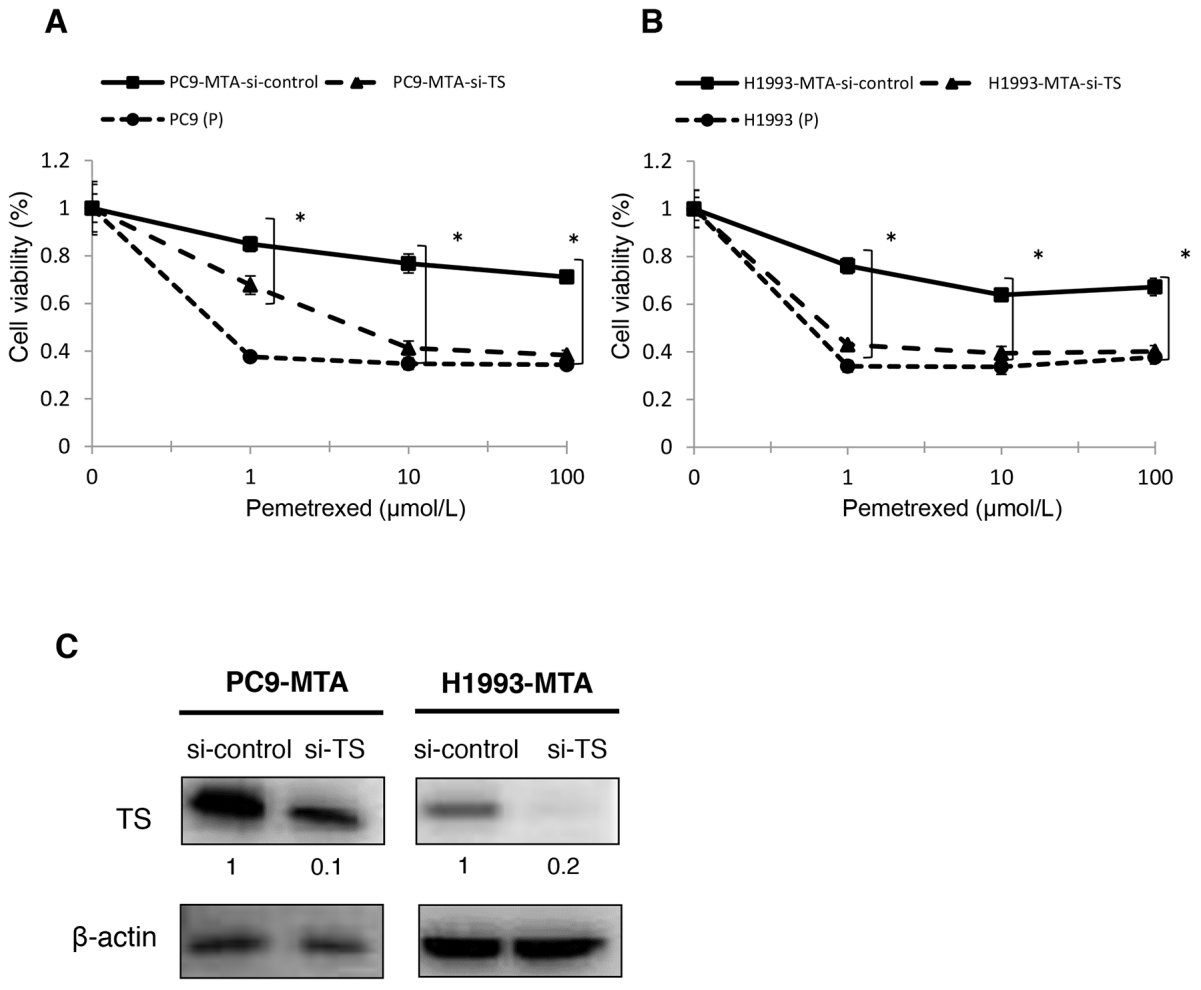
Effects of TS knockdown in pemetrexed-resistant lung cancer cells The expression of TS in PC9-MTA and H1993-MTA cells was inhibited by siRNA, and the sensitivity to pemetrexed was tested using WST assays. **(A)** Sensitivity to pemetrexed was measured in PC9-MTA cells transfected with siRNA targeting control or TS (si-control or si-TS) and the parental PC9 cells (P). **(B)** Sensitivity to pemetrexed was measured in H1993-MTA cells transfected with siRNA (si-control or si-TS) and the parental H1993 cells. The error bars in each graph represent the standard error of the value obtained in the experiments performed in triplicate. **(C)** TS expression as determined by the Western blot analyses in PC9-MTA cells and H1993-MTA cells transfected with si-TS or si-control.

To evaluate whether the elevated expression of FGF2 and TS was functionally important in acquiring resistance to pemetrexed, we tested whether the knockdown of either factor would restore pemetrexed sensitivity in PC9-MTA and H1993-MTA cells (Figure 2A, 2B, 3A and 3B). Following inhibition of FGF2 or TS by small interfering RNA (siRNA), their expression was confirmed at the protein level by Western blot analysis (Figure 2C, 3C). When the pemetrexed-resistant cells were transfected with si-FGF2, the sensitivity to pemetrexed was partially restored in both PC9-MTA and H1993-MTA (Figure 2A and 2B), whereas pemetrexed sensitivity was restored to a greater degree in both cell lines upon siRNA-mediated TS inhibition, with the level in H1993-MTA cells comparable to that of the parental H1993 cells (Figure 3A and 3B).

### Inhibition of FGF2 alters the expression of signal transduction molecules in the pemetrexed-resistant lung cancer cell lines

**Figure 4:**
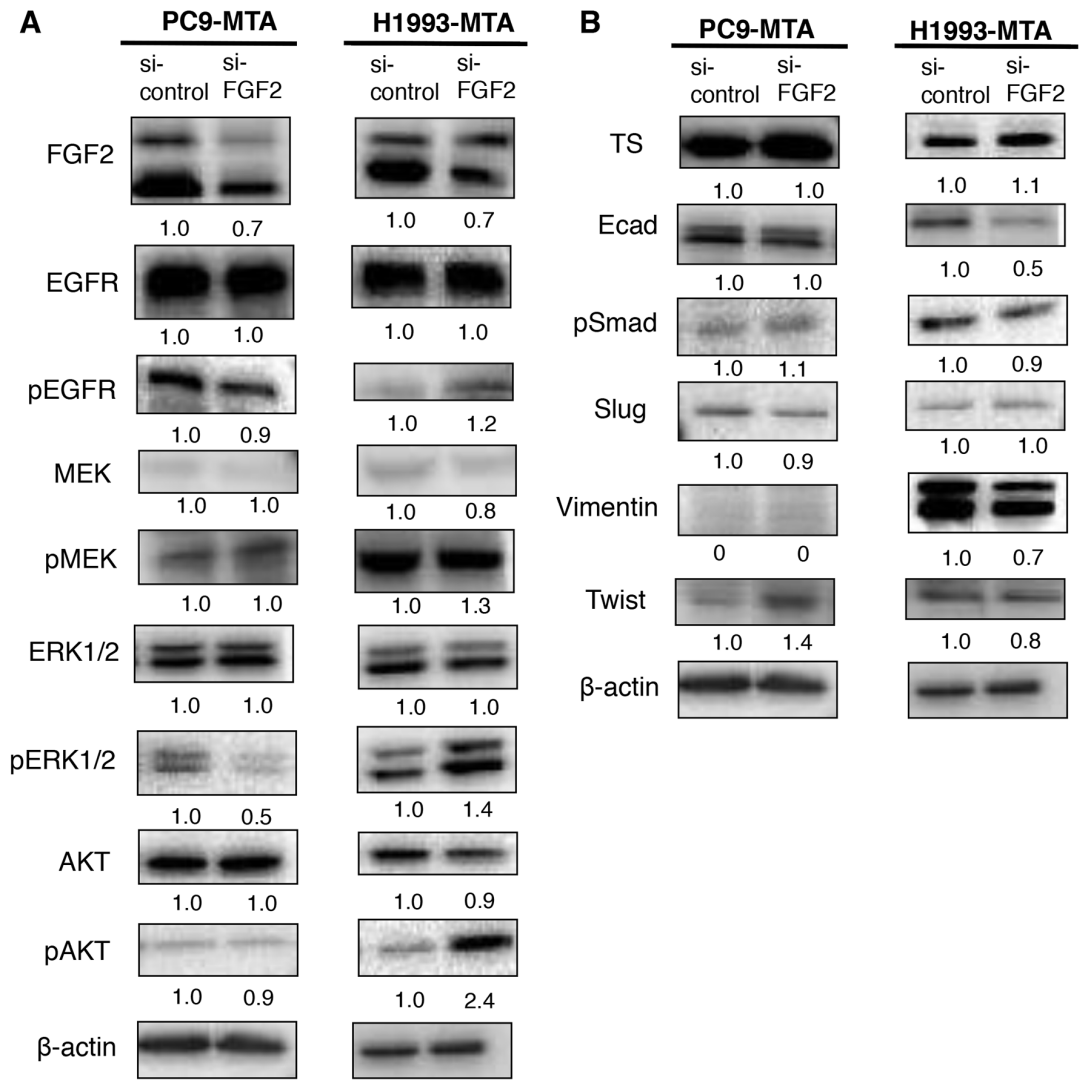

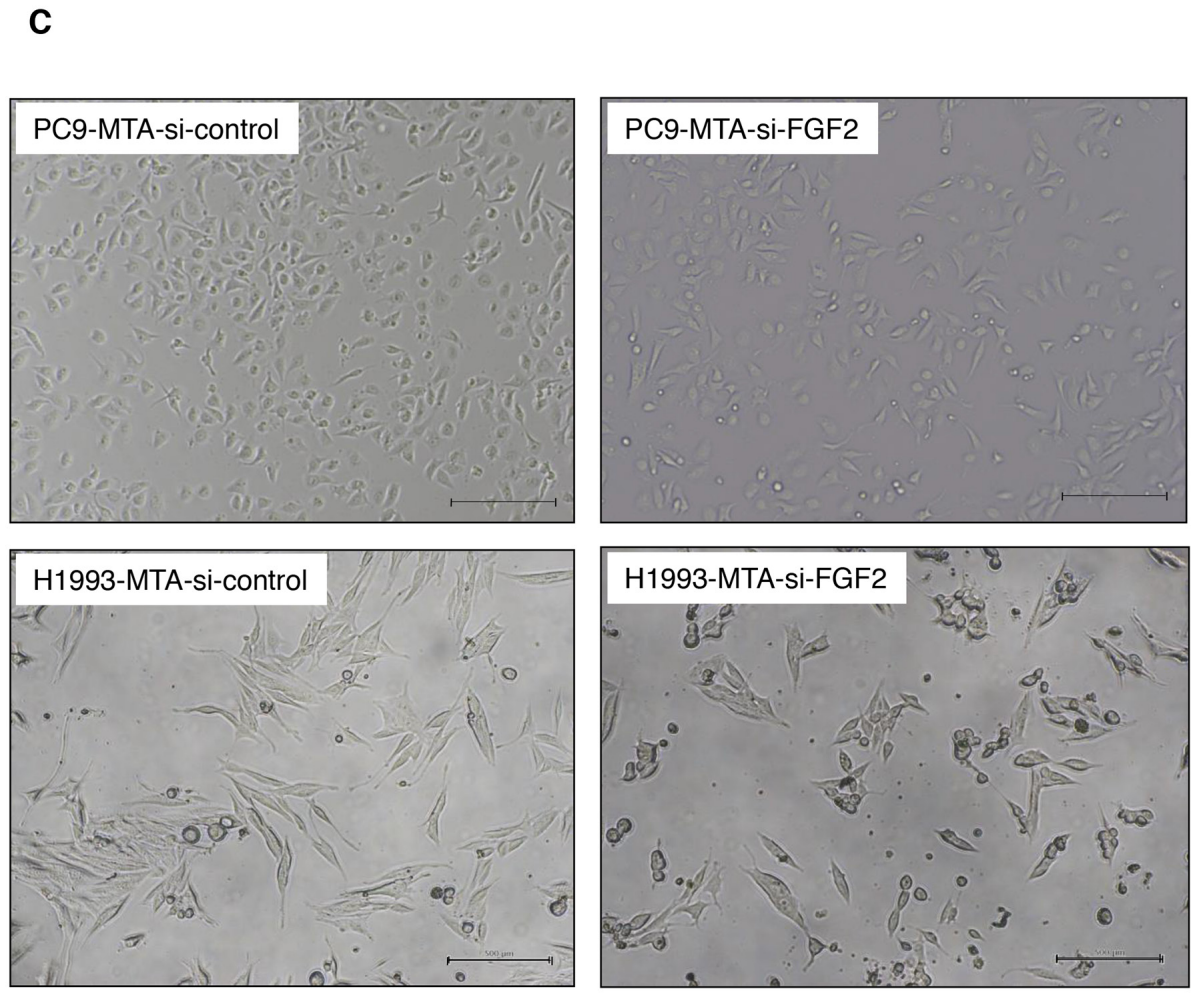
Effects of FGF2 knockdown on signaling pathway molecules and EMT marker proteins in the parental and pemetrexed-resistant lung cancer cells **(A)** Western blot analyses of expression of total or phosphorylated forms (pEGFR, pMEK, pERK, and pAKT) of signaling molecules in the PC9-MTA and H1993-MTA cells transfected with control siRNA (si-control) or siRNA targeting FGF2 (si-FGF2). β-actin was used as a loading control. The experiments were repeated independently at least three times, and one representative blot is provided in the figures. The quantitative numbers of relative expression levels corrected by β-actin were demonstrated below the picture of the blots. **(B)** Western blot analyses of expression of TS and EMT marker proteins in the PC9-MTA and H1993-MTA cells transfected with si-control or si-FGF2. **(C)** Morphological findings of PC9-MTA and H1993-MTA cells transfected with si-control or si-FGF2. Scale bars = 500 μm.

We next evaluated whether the inhibition of FGF2 would affect the expression or phosphorylation of the signaling molecules in the MAPK/ERK and PI3K/AKT pathways or the expression of TS by Western blotting (Figure 4A). In PC9-MTA cells, the phosphorylation of ERK1/2 was decreased when the cells were transfected with si-FGF2. Thus, the inhibition of FGF2 reversed the phosphorylation of the signaling proteins in the PI3K-AKT pathway in PC-MTA cells, suggesting that the enhancement of FGF2 expression in PC9-MTA cells activated kinase pathway signaling concomitant with pemetrexed resistance. Conversely, the phosphorylation of EGFR, ERK1/2, and AKT, which was inhibited in H1993-MTA cells, was restored by the inhibition of FGF2. These results suggested that FGF2 enhancement in the development of resistance to pemetrexed might inhibit kinase pathway signaling by inhibiting the phosphorylation of EGFR in H1993-MTA cells. Thus, the effects induced by FGF2 were different in each lung cancer cell line, although the expression of FGF2 was similarly upregulated during the development of pemetrexed-resistance. This indicated that although pemetrexed sensitivity was restored in both PC9-MTA and H1993-MTA cells upon transfection with si-FGF2, the underlying mechanisms of restoration likely differ in the two cell lines. Finally, the expression of TS was not affected by the transfection of si-FGF2 in either PC9-MTA or H1993-MTA cells (Figure 4B), which indicated that the upregulation of TS might be induced by mechanisms other than those regulated by increased expression of FGF2 in these lung cancer cells.

### Inhibition of FGF2 reverses the phenotype from mesenchymal to epithelial in H1993-MTA, but not PC9-MTA cells

As EMT was observed in H1993 cells during the development of pemetrexed-resistance, we tested whether the inhibition of FGF2 would alter the phenotype of the pemetrexed-resistant cells (Figure 4B and 4C). In H1993-MTA cells, the expression of vimentin was decreased by the introduction of si-FGF2; however, the alteration of the other markers was not distinct. Moreover, the inhibition of FGF2 partially reversed the morphology of H1993-MTA cells from a spindle-shaped to an epithelial morphology (Figure 4C). However, distinct effects on the expression of EMT markers were not observed except for a slight increase of expression of Twist in the PC-MTA cells. In addition, no morphological change was observed by the inhibition of FGF2 in PC9-MTA cells. These results also demonstrated that the upregulated FGF2 might act differently in each pemetrexed-resistant cell line.

### Restoration of pemetrexed sensitivity by PD173074 in pemetrexed-resistant lung cancer cells

**Figure 5:**
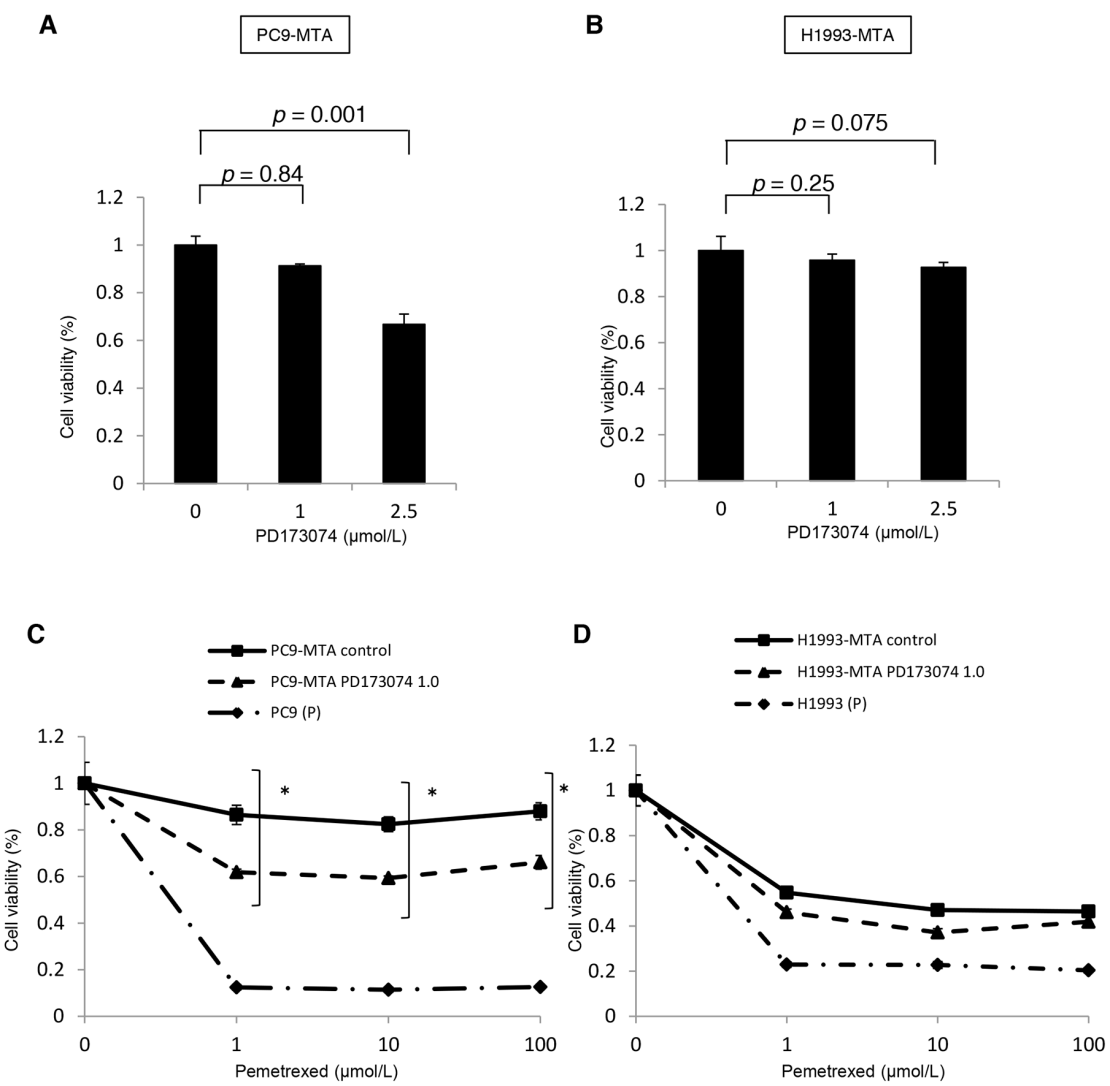

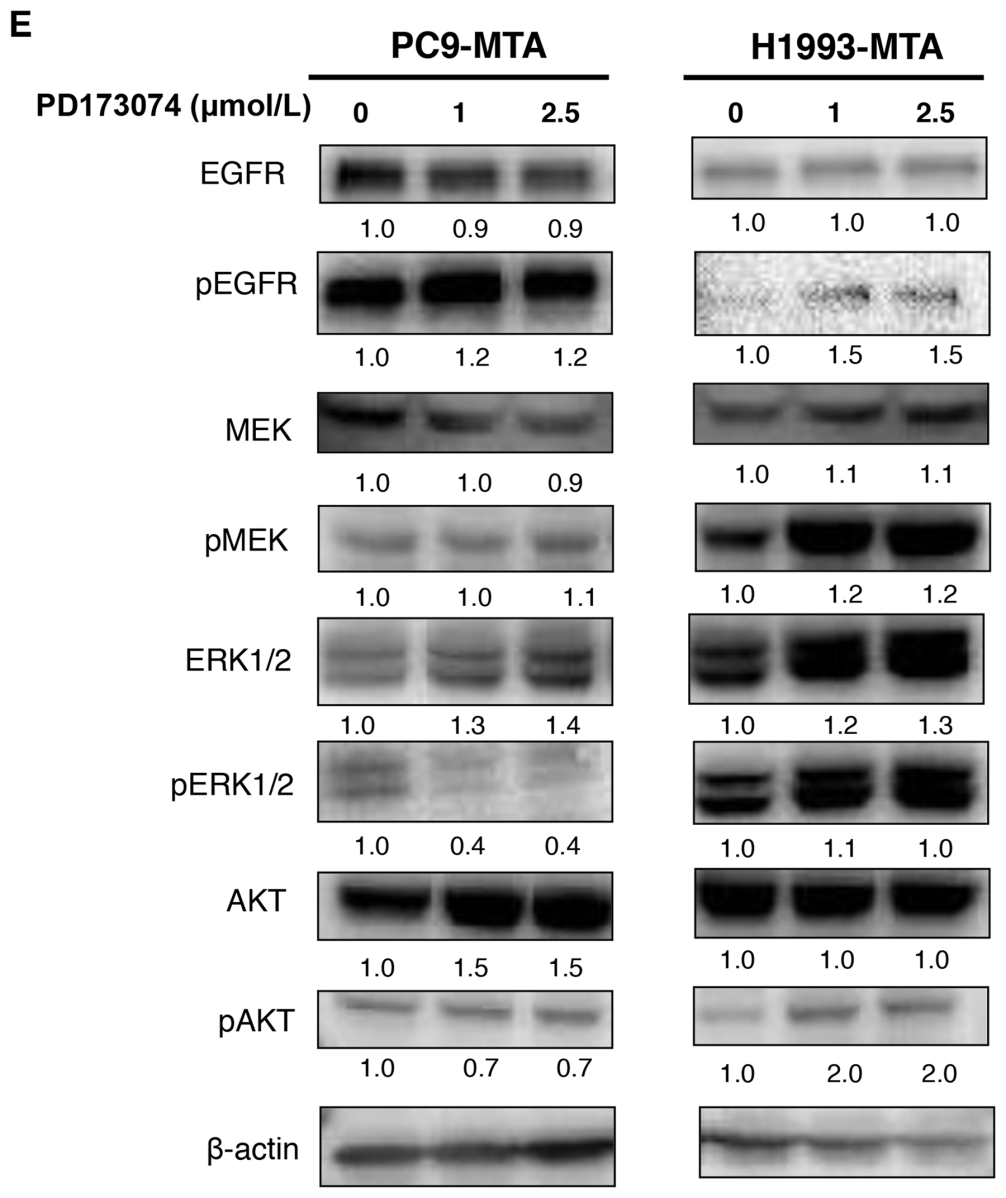

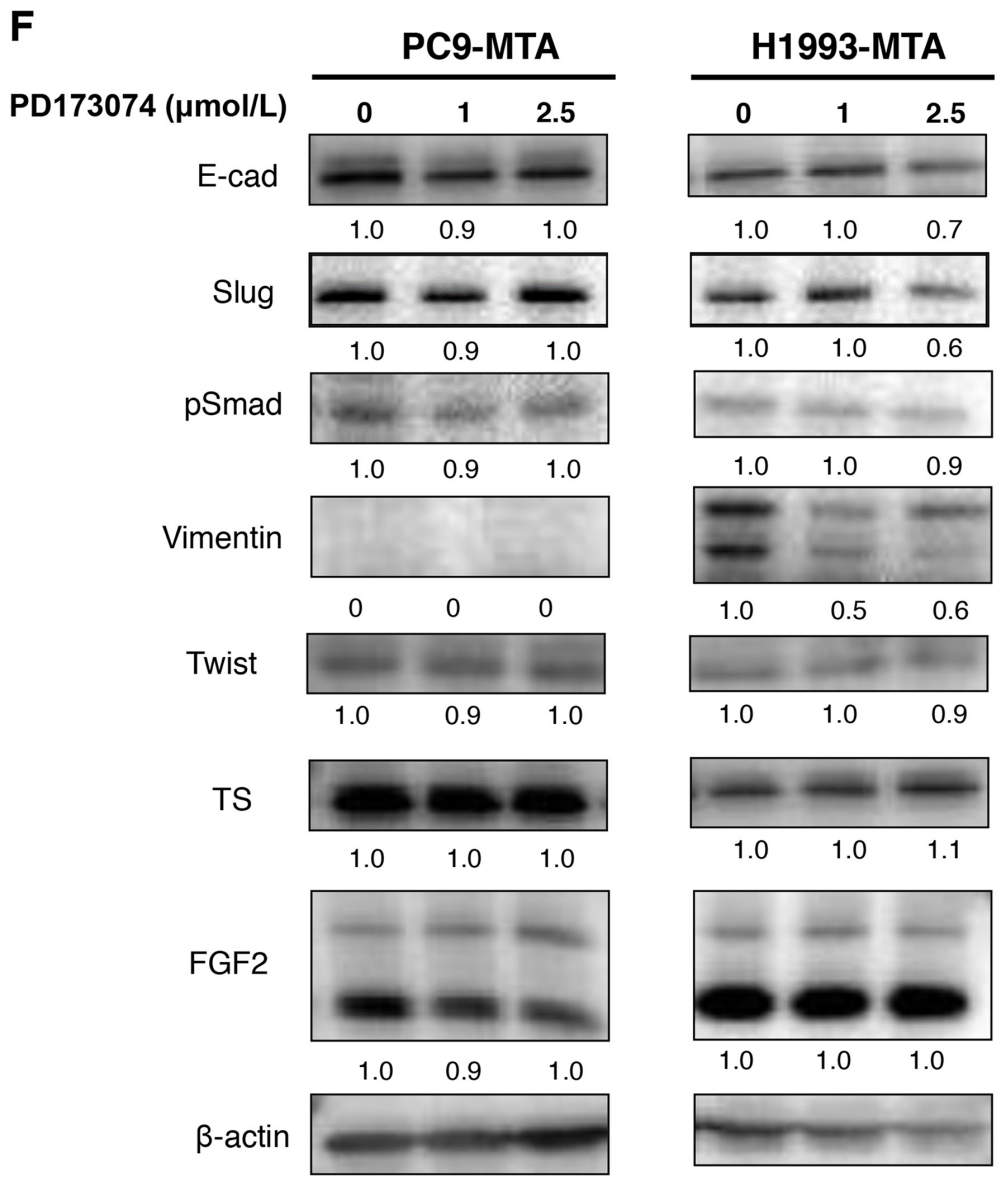
Effect of an FGFR1 inhibitor (PD173074) on the sensitivity to pemetrexed in pemetrexed-resistant lung cancer cells **(A, B)** Effects of PD173074 on the growth of pemetrexed-resistant PC9 PC9-MTA cells and H1993-MTA cells were measured by WST assay. The cells were cultured for 72 h in the presence of 0 to 5 μM PD173074. **(C)** Effects of PD173074 on the sensitivity to pemetrexed in the parental PC9 (P) and the PC9-MTA cells were measured by WST assay. **(D)** Effects of PD173074 on the sensitivity to pemetrexed in the parental H1993 (P) and H1993-MTA cells were measured by WST assay. The error bars in each graph represent the standard error of the value obtained in the experiments performed in triplicate. **(E)** The effects of PD173074 on the expression of total or phosphorylated forms of signaling molecules in the MAPK/ERK and PI3K/AKT pathways, EMT markers, and TS expression were analyzed by Western blotting in PC9-MTA and H1993-MTA cells. β-actin was used as a loading control. The experiments were repeated independently at least three times, and one representative blot is provided in the figures. The quantitative numbers of relative expression levels corrected by β-actin were demonstrated below the picture of the blots. **(F)** The effects of PD173074 on the expression of total or phosphorylated forms of signaling molecules in the MAPK/ERK and PI3K/AKT pathways, EMT markers, and TS expression were analyzed by Western blotting in PC9-MTA and H1993-MTA cells. β-actin was used as a loading control. The experiments were repeated independently at least three times, and one representative blot is provided in the figures. The quantitative numbers of relative expression levels corrected by β-actin were demonstrated below the picture of the blots.

Next, to evaluate whether inhibition of the FGF2-FGFR1 signaling pathway might restore pemetrexed sensitivity, we tested the effect of PD173074, a specific FGFR inhibitor, on pemetrexed sensitivity and signal transduction in the PC9-MTA and H1993-MTA cells. PD1730174 showed a dose-dependent effect on growth inhibition in both PC9-MTA and H1993-MTA cells (Figure 5A, 5B). Specifically, although 1 μM PD173074 showed only a slight growth inhibition in both cell lines, the altered expression of signaling molecules was confirmed by Western blot (Figure 5E). Therefore, we tested the effect of 1 μM PD1730174 on the sensitivity to pemetrexed. In the WST assay, pemetrexed sensitivity was partially restored by 1 μM PD1730174 in PC9-MTA cells, whereas only marginal restoration was obtained in H1993-MTA cells (Figure 5C, 5D).

To investigate whether PD173074 affects kinase pathway signaling, we evaluated the expression or phosphorylation of the downstream signaling molecules by Western blotting (Figure 5E). Incubation of PC9-MTA cells with PD173074 for 24 h inhibited ERK1/2 phosphorylation, whereas the status of EGFR and the other signaling molecules was not changed. In contrast, the phosphorylation of EGFR, MEK, ERK1/2, and AKT was enhanced by the addition of 1 μM PD173074 in H1993-MTA cells. Thus, inhibition of the FGF2-FGFR pathway by PD173074 inhibited the MAPK/ERK pathway in PC9-MTA cells, whereas it activated the MAPK/ERK and PI3K/AKT pathways in H1993-MTA cells.

The addition of PD173074 did not alter the expression of EMT molecular marker proteins in PC9-MTA cells (Figure 5F) but decreased the expression of vimentin and Slug in H1993-MTA cells. Conversely, FGF2 and TS expression was not affected by PD173074 in either cell line. Thus, the effects induced by FGFR inhibitor on the FGF2-FGFR1 pathway were consistent with those obtained by siRNA-mediated FGF2 inhibition in these pemetrexed-resistant cells.

### Restoration of pemetrexed sensitivity by dual inhibition of FGF2 and TS in pemetrexed-resistant lung cancer cells

**Figure 6:**
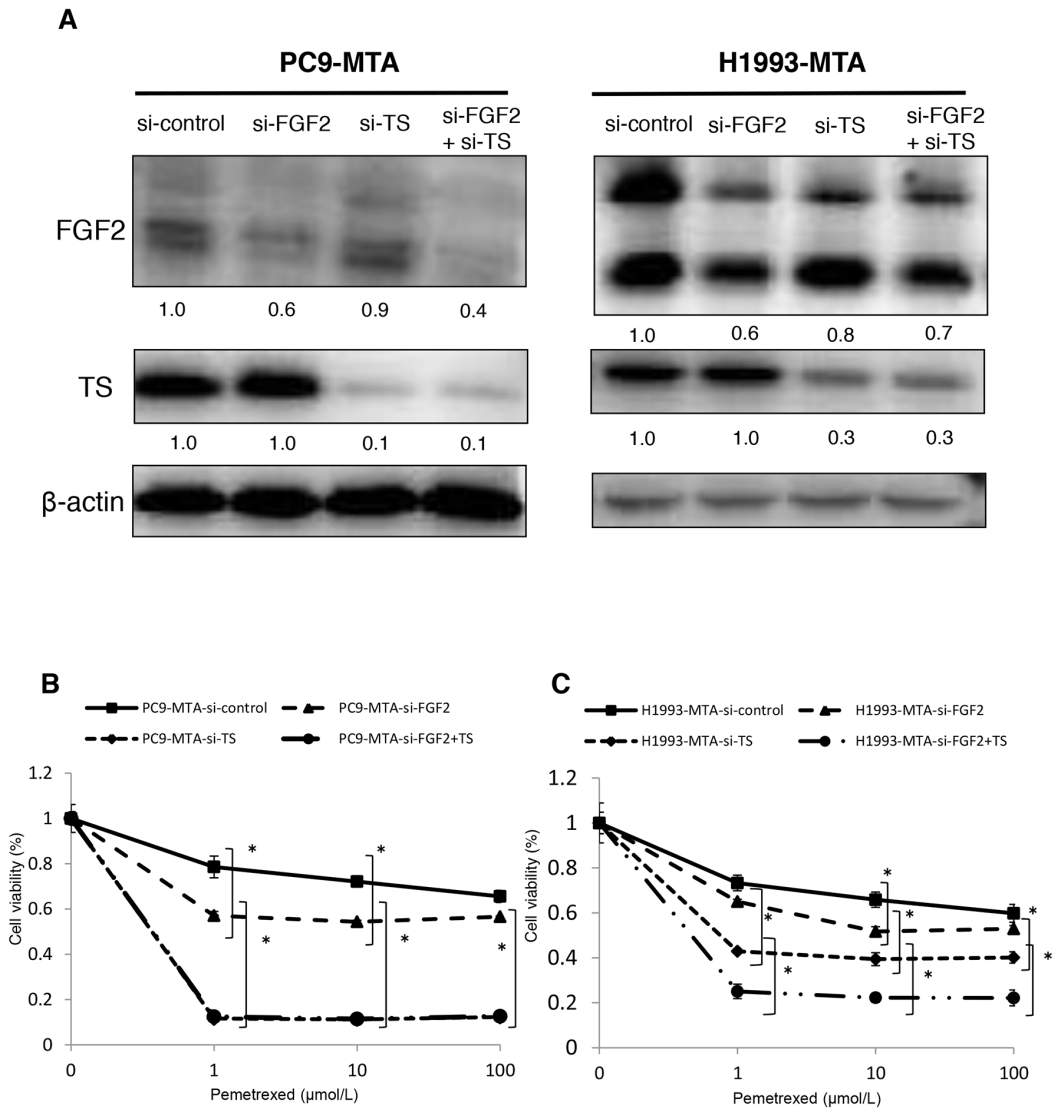
Effects of individual or dual knockdown of FGF2 and TS on sensitivity to pemetrexed in the pemetrexed-resistant lung cancer cells **(A)** FGF2 and TS expression as determined by Western blot analyses in the PC9-MTA cells and H1993-MTA cells transfected with control siRNA (si-control), siRNA targeting FGF2 (si-FGF2), siRNA targeting TS (si-TS), or both (si-FGF2 + TS). β-actin was used as a loading control. **(B, C)** Sensitivity to pemetrexed was measured in PC9-MTA **(B)** or H1993-MTA **(C)** cells transfected with si-control, si-FGF2, si-TS, or si-FGF2 + TS. The error bars in each graph represent the standard error of the value obtained in the experiments performed in triplicate.

Next, we tested whether the dual inhibition of FGF2 and TS by siRNAs would restore the pemetrexed sensitivity additively in the PC9-MTA and H1993-MTA cells (Figure 6). The expression of TS was not inhibited by transfection with the FGF2-targeting siRNA, and FGF2 was not inhibited by transfection with the TS-targeting siRNA. When these sublines were transfected with respective siRNAs targeting both FGF2 and TS, the expression levels of both proteins were inhibited to the same level as when the cells were transfected with either siRNA alone (Figure 6A); thus, their respective expression was not inhibited by transfection with siRNA targeting the other factor. Notably, the inhibition of both FGF2 and TS restored the pemetrexed sensitivity additively in H1993-MTA cells. However, although the inhibition of either FGF2 or TS restored the pemetrexed sensitivity in PC9-MTA cells, the inhibition of TS alone restored the pemetrexed sensitivity to a level equivalent to that induced by the dual inhibition of FGF2 and TS (Figure 6B, 6C). Although these results indicated that both FGF2 and TS might be involved in the development of pemetrexed resistance in lung cancer cells, TS might contribute to the emergence of resistance to a greater extent than FGF2.

### Restoration of pemetrexed sensitivity by inhibition of ZEB1 in pemetrexed-resistant H1993 cells

**Figure 7:**
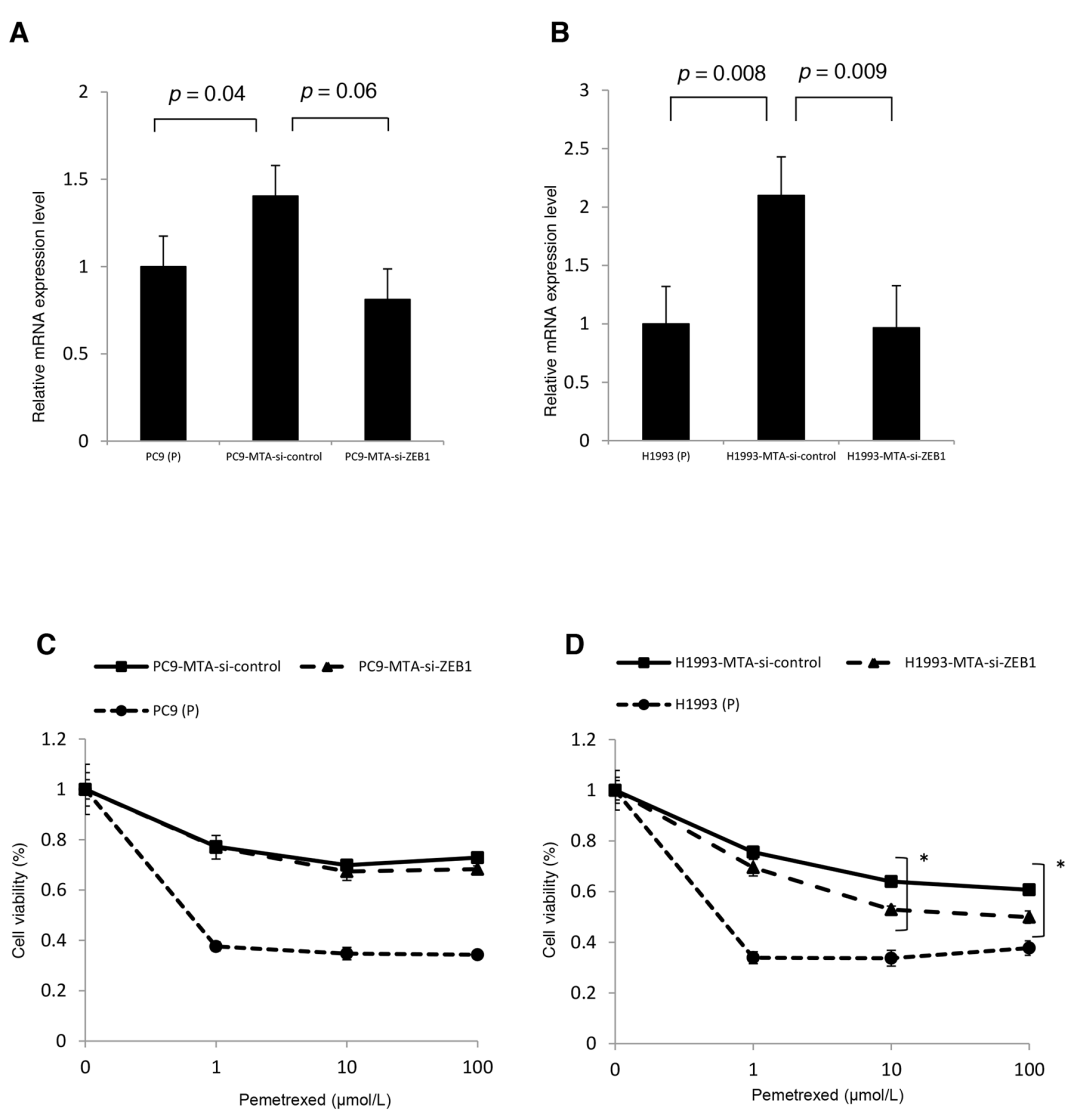

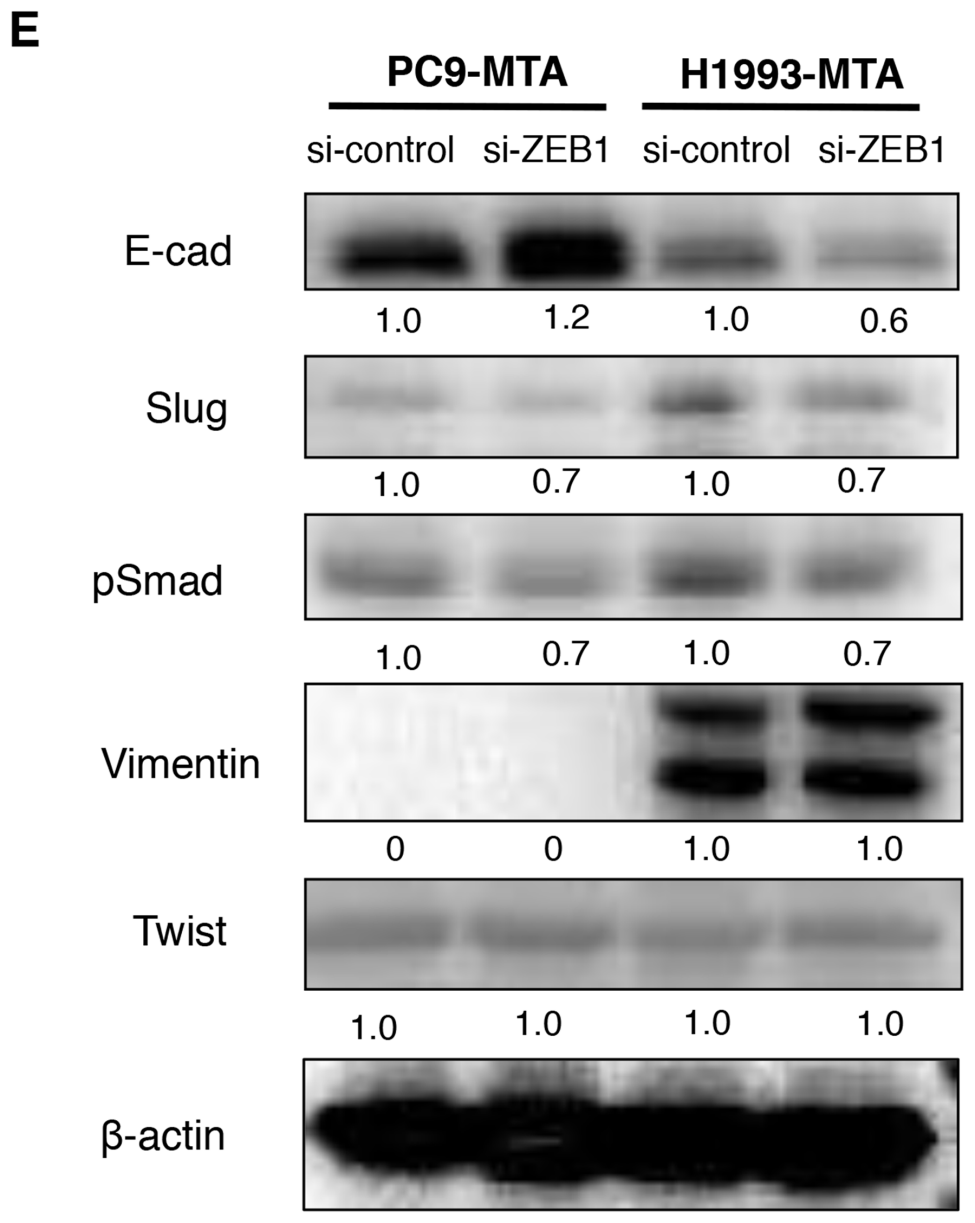
Effects of ZEB1 knockdown on the sensitivity to pemetrexed in the pemetrexed-resistant lung cancer cells **(A, B)** *ZEB1* expression was quantitated by real-time RT-PCR in the parental PC9 cells (P) and PC9-MTA cells (A) and the parental H1993 (P) cells and H1993-MTA cells (B) transfected with control si-RNA (si-control) and siRNA targeting ZEB1 (si-ZEB1). **(C, D)** Sensitivity to pemetrexed was measured in parental (P) and PC9-MTA cells **(C)** and in parental H1993 (P) and H1993-MTA cells **(D)** transfected with each si-RNA (si-control and si-ZEB1) using the WST assay. The error bars in each graph represent the standard error of the value obtained in the experiments performed in triplicate. **(E)** Western blot analysis of the expression of EMT marker proteins in PC9-MTA and H1993-MTA cells transfected with si-control or si-ZEB1. β-actin was used as a loading control.

To evaluate the association between EMT and pemetrexed resistance, we tested the effects of siRNA-mediated ZEB1 knockdown on the sensitivity to pemetrexed in PC9-MTA and H1993-MTA cells. RT-PCR demonstrated elevated *ZEB1* mRNA expression in both sublines compared to that in their respective parental cells (Figure 7A, 7B). In comparison, when the pemetrexed-resistant sublines were transfected with ZEB1-targeting siRNA, the sensitivity to pemetrexed was partially restored in H1993-MTA cells, but not in PC9-MTA cells (Figure 7C and 7D). With regard to the expression of EMT-related proteins, a decrease in Slug and pSmad levels was induced in H1993-MTA cells when ZEB1 expression was inhibited by ZEB1-targeting siRNA, although the expression of vimentin was not altered (Figure 7E). On the other hand, the expression of none of the EMT molecular marker proteins was altered by the inhibition of ZEB1 in PC9-MTA cells. These results supported the possibility that the mesenchymal phenotype observed in H1993-MTA cells was involved partly in decreased sensitivity to pemetrexed.

### Pemetrexed-resistant sublines do not show cross-resistance to gefitinib

In a previous study, Terai et al. established a gefitinib-resistant subline in the PC9 cells and reported that the expression of FGF2 was increased in this subline; they also showed that increased expression of FGF2 conferred this resistance to gefitinib, an EGFR-TKI [14]. In the present study, as we found that the expression of FGF2 was increased in the pemetrexed-resistant PC9 cells, we examined whether the PC9-MTA and H1993-MTA cells might demonstrate cross-resistance to gefitinib. However, neither subline exhibited cross resistance to gefitinib. Rather, the sensitivity to gefitinib was slightly increased in each subline compared to their respective parental cells (Supplementary Figure 2).

## DISCUSSION

In this study, we revealed two novel findings regarding pemetrexed resistance in lung cancer. First, the expression of both FGF2 and FGFR1 was markedly elevated in the pemetrexed-resistant lung cancer sublines derived from two different lung cancer cell lines originated from adenocarcinoma, whereas the inhibition of the FGF2-FGFR1 pathway using FGF2-targeting siRNA or the specific FGFR inhibitor PD173074 partially restored the pemetrexed sensitivity. Second, the mechanisms of pemetrexed resistance conferred by upregulation of the FGF2-FGFR1 pathway might differ in each cancer cell line, with the induced alteration of cellular functions potentially dependent on the innate molecular features of lung cancer cells such as the presence or absence of *EGFR* mutation, which might in turn affect the function of downstream signaling molecules in the kinase pathways of these cells. TS was also markedly elevated and substantively contributed to the development of resistance to pemetrexed in the generated lung cancer sublines, similar to previous report [4, 6, 17]. However, the pemetrexed resistance conferred by FGF2-FGFR1 pathway upregulation was considered to be independent from that mediated by TS, as the inhibition of either FGF2 or TS by siRNA had no effect on expression of the other factor. To the best of our knowledge, this is the first report to demonstrate the involvement of the FGF2-FGFR1 pathway in the development of pemetrexed resistance in lung cancer cells.

FGF2 has been reported to be involved in the resistance to anticancer agents, both cytotoxic chemotherapeutic and molecular-targeted, in various malignant tumors including lung cancer [14, 18, 19, 20, 21, 22, 23]. With regard to the former, it has been reported that the overexpression of FGF2 is correlated with chemoresistance in several malignant tumors [24, 25, 26, 27, 28, 29]. For example, the transduction of *FGF2* cDNA into NIH 3T3 cells conferred resistance to cisplatin, etoposide, and 5-FU [30, 31]. In the HT1376 bladder cancer cell line, transduction of *FGF2* cDNA also led to resistance to cisplatin [32]. Recently, Makondi et al. demonstrated the enhancement of tumor responsiveness to irinotecan upon MAPK signal transduction pathway inhibition through the targeting of FGF2 in colorectal cancer [22]. In addition, Gan et al. indicated that FGF2 expression was a strong predictor of paclitaxel resistance in clinical specimens of bladder tumors. Thus, the increased expression of FGF2 appears to confer resistance to various cytotoxic chemotherapeutic agents in cancer cells, which is consistent with our results obtained for pemetrexed-resistant lung cancer cells.

Recent studies have revealed that the activation of alternative pathways constitutes one of the mechanisms involved in the acquisition of resistance to EGFR-TKIs in NSCLC lines harboring *EGFR* mutations including PC9 [13, 14, 16, 33, 34]. Terai et al. demonstrated the upregulation of the FGF2-FGFR1 pathway together with the activation of ERK1/2 in gefitinib resistant PC9 cells in which EGFR phosphorylation was repressed, along with the restoration of gefitinib sensitivity upon FGF2 knockdown [14]. Azuma et al. demonstrated downregulation of EGFR family proteins and their phosphorylated molecules together with an alternative upregulation of FGFR1 and its ligand FGF2 in PC9 cells resistant to afatinib, an irreversible inhibitor of the ErbB family [16]. These studies demonstrated that activation of the FGF2-FGFR autocrine loop has a compensatory role in promoting the survival and growth of EGFR-TKI resistant cells, suggesting the close interaction of EGFR- and FGFR-driven cell growth or signaling pathways. In the present study, the increased expression of both FGF2 and FGFR1 together with the activation of pERK were observed in PC9 cells after long exposure to pemetrexed; however, the inhibition of EGFR was not observed in the PC9-MTA cells. The results of the present study thus suggested that inhibition of the EGFR signaling pathway might not be necessary to activate the FGF2-FGFR1 autocrine loop, which may instead be induced as a response to the additional stress applied to the PC9 lung cancer cells upon pemetrexed exposure.

The activation of FGF2-FGFR-driven signaling pathways by acquired resistance to EGFR-TKIs such as gefitinib and afatinib has been reported, primarily in NSCLC cells harboring *EGFR* mutation [14, 16]. However, there have been few reports describing the role of the FGF2-FGFR1 pathway in NSCLC cells with wild-type *EGFR* despite the demonstration that activation of this pathway represents an important mechanism of growth in such cell lines [12, 13]. Marek et al. demonstrated activation of FGF2-FGFR in some gefitinib-insensitive NSCLC cells with wild-type *EGFR* [13]. Notably, we observed the inhibition of EGFR phosphorylation together with the inhibition of downstream signaling molecules in the MAPK/ERK and PI3K/AKT pathways in H1993-MTA cells, which carry wild-type *EGFR*. Furthermore, although both FGF2 and FGFR1 demonstrated a marked increase in H1993-MTA cells, the downstream signaling molecules in the MAPK/ERK and PI3K/AKT pathways remained inhibited in these cells, which cannot readily be explained by the activation of alternative bypass track theory presumed in the *EGFR* mutant NSCLC cell lines [14, 34]. Our data instead suggest a possibility that the downstream signaling alteration caused by FGF2-FGFR1 pathway activation might interact closely with EGFR pathway status, which depends largely on the presence or absence of *EGFR* mutations as well as the statuses of other receptor tyrosine kinases that are also considered to be involved in lung oncogenesis [35].

Alternatively, EMT has been demonstrated to be one of the mechanisms underlying resistance to cytotoxic agents such as cisplatin, oxaliplatin, and taxanes in various cancers both *in vitro* and *in vivo*. For example, EMT markers were upregulated in ovarian cancer cell lines resistant to paclitaxel [36], and Yang et al. reported that morphological changes suggestive of EMT were observed in colon cancer cells resistant to oxaliplatin [37]. EMT is also reportedly related to docetaxel resistance in patients with prostate cancer [38]. Moreover, Shintani et al. demonstrated the upregulation of EMT markers in those patients with NSCLC showing resistance to cisplatin-based chemotherapy [39], which is consistent with findings that knockdown of Snail caused increased NSCLC cell sensitivity to cisplatin [40]. In addition, FGF signaling has been demonstrated to be involved in the regulation of EMT by WNT, Notch, Hedgehog, and TGFβ signaling cascades [41].

In the present study, a phenotype switch from epithelial to mesenchymal was induced by long exposure to pemetrexed in H1993 cells; however, not all marker proteins were typically upregulated or downregulated upon acquisition of pemetrexed resistance. Furthermore, inhibition of the FGF2-FGFR1 pathway by FGF2-targeting siRNA or anti-FGFR1 antibody reduced the expression of mesenchymal markers together with the partial restoration of pemetrexed sensitivity in H1993-MTA cells. In addition, the inhibition of ZEB1 by siRNA also partially restored the sensitivity to pemetrexed. These findings suggest that a phenotype transition to mesenchymal cell features mediated by the FGF2-FGFR1 pathway might be involved in the development of pemetrexed resistance in H1993 cells, although the role of this pathway in the pemetrexed resistance was not as substantial as that of TS.

Conversely, the alteration of EMT marker protein expression, except for a slight increase of E-cadherin and ZEB1, was not observed in PC9-MTA cells despite a marked increase of both FGF2 and FGFR1 expression. Recently, Azuma et al. demonstrated that an EGFR-TKI-, afatinib-resistant subclone of PC9, in which EGFR phosphorylation was inhibited, demonstrated EMT characteristics relative to the afatinib-sensitive parental PC9 line [16]. Similarly, in the H1993-MTA cells established in our study, the inhibition of EGFR phosphorylation was also clearly observed. Our findings together with those by Azuma et al. therefore indicate that inhibition of the EGFR pathway together with activation of the FGF2-FGFR1 pathway might thus be required for the transition of phenotype from epithelial to mesenchymal. However, the information provided to date by both the previous and our current data cannot fully explain the paradoxical phenomena observed between PC9-MTA and H1993-MTA cells in the present study, indicating that the effects of FGF2-FGFR pathway activation on cellular phenotype as well as on signal transduction might depend on the innate molecular characteristic of lung cancer cells.

Taken together, our results suggest that the possible mechanisms of pemetrexed resistance in PC9 and H1993 cells may be summarized as follows. (i) For both cell lines, pemetrexed resistance could largely be attributed to the enhanced expression of TS and partly to the upregulation of the FGF2-FGFR pathway. (ii) In PC9 cells, which carry *EGFR* exon 19 deletion, upregulation of the FGF2-FGFR1 pathway activated the MAPK/ERK pathway, conferring the resistance to pemetrexed without altering signaling via the EGFR pathway. (iii) In H1993 cells, which carry wild-type *EGFR*, EGFR pathway inhibition and FGF2-FGFR1 pathway enhancement were simultaneously induced in the pemetrexed-resistant subline. Moreover, the EMT mediated by FGF2-FGFR pathway activation was specifically associated with pemetrexed resistance.

In the present study, the PC9-MTA subline did not show a cross resistance to gefitinib despite a higher expression of both FGF2 and FGFR, which Terai et al. considered as indicative of a compensatory pathway in a gefitinib-resistant PC9 subclone [14]. Thus, our data suggest that the upregulation of the FGF2-FGFR1 pathway in *EGFR* mutation-positive lung cancer cells does not always confer resistance to gefitinib; conversely, the alteration of cellular function induced by FGFR pathway activation might instead be affected by the status of a network consisting of various signaling cascades such as other receptor tyrosine kinases, WNT, Notch, and TGFβ in each cancer cell [41]. However, our data together with that of previous studies suggest that the upregulation of the FGF2-FGFR1 pathway might occur in response to the cellular stress generated by the agents regardless of whether they are chemotherapeutically or molecularly targeted.

There were some limitations in this study. Although we utilized two NSCLC cell lines with different *EGFR* status, additional cell lines with different molecular features should be tested to confirm the universality of involvement of the FGFR pathway in the development of pemetrexed resistance in lung cancer. In addition, the data in this study were obtained from *in vitro* experiments alone. As the cancer microenvironment is important for tumor growth and the development of chemoresistance, further studies including *in vivo* analysis and incorporating clinical materials should be performed.

In conclusion, our data demonstrated that the FGF2-FGFR1 pathway is upregulated in the pemetrexed-resistant NSCLC cells lines and that knockdown of FGF2 by siRNA or the addition of an anti-FGFR1 antibody could partially restore the sensitivity to pemetrexed without altering TS expression. Thus, we demonstrated that the enhancement of the FGF2-FGFR1 pathway constitutes a novel mechanism of pemetrexed resistance in NSCLC cells, although the effect of resistance mediated by the FGF2-FGFR1 pathway is not as strong as that by TS. Recent studies have demonstrated the therapeutic potential of inhibition of the FGFR pathway in many cancers including lung cancer [42, 43]. Thus, although the mechanisms of activation of the FGF2-FGFR1 pathway during the development of pemetrexed resistance remains to be further studied, we expect that FGFR-targeted therapy in combination with pemetrexed may represent a novel therapeutic strategy to treat patients with NSCLC.

## MATERIALS AND METHODS

### Cell culture and treatment agents

The two NSCLC cell lines, PC9 [*EGFR* exon 19 deletion (delE746-A750) and H1993 [*EGFR* wild-type] were kindly donated by Dr. Ono at Kyusyu University, Japan. All cell lines were cultured in RPMI-1640 with 10% fetal bovine serum at 37°C and 5% CO_2_. Pemetrexed-resistant cell lines were established in our institute by continuous exposure with stepwise increase in the concentration of pemetrexed for over six months, with the medium replaced every 3 days. The cultured cells were subcultured by trypsinization when the cells reached 70–80% confluency. Several pemetrexed-resistant clones were selected for PC9 and H1993 by limiting dilution method, with a single representative clone for each line used in the experiments. Pemetrexed was purchased from LC Laboratories (Woburn, MA, USA), and PD173074 and gefitinib were purchased from Selleckchem (Houston, TX, USA).

### WST assay

The growth inhibitory effects of pemetrexed were measured using the WST assay (Wako Chemicals, Osaka, Japan) according to the manufacturer’s instructions. Briefly, 4 × 10^3^ cells were cultured in 96-well plates and allowed to attach for 24 h. Then, the cells were treated with pemetrexed or gefitinib at variable concentrations, and cultured for 72 h in the experiment determining the IC_50_ for the pemetrexed-resistant cells and 72 h in the experiments involving transfection with siRNAs. Then, 10 μL WST-8 solution was added to each well and the plates were incubated at 37°C for another 3 h. Absorbance was measured at 450 and 640 nm using the SoftMax Pro (Molecular Devices, Tokyo, Japan) and the cell viability was determined. Each experiment was independently performed and repeated at least three times. As an exception, 2 × 10^3^ cells were cultured in 96-well plates with pemetrexed and PD173074 in each concentration when treated with PD173074; after additional cultured for 72 h, absorbance was measured as described above.

### Total RNA extraction and quantitative real-time RT-PCR

Total RNA was extracted using an RNeasy Mini kit (Qiagen, Alameda, CA, USA) according to the manufacturer’s instructions. TaqMan Gene Expression Assays for *FGFR1* (#Hs00241111_m1), *ZEB1* (#Hs01566408_m1), and β-actin (#Hs99999903_m1) were purchased from Applied Biosystems (Carlsbad, CA, USA), and mRNA levels were quantified in triplicate using the Applied Biosystems 7300 Real-Time PCR system.

### Microarray analysis

Total RNA from parental PC9 and PC9-MTA cells and H1993 and H1993-MTA cells were extracted using an RNeasy Mini kit (Qiagen) according to the manufacturer’s instructions. Microarray analysis using an Agilent SurePrint G3 Human GE v2 8 × 60K Microarray (Design ID: 039494) was performed at DNA Chip Research Inc. (Tokyo, Japan).

### Western blotting

Proteins were isolated from cells as previously described, and were then used in the Western blot analyses (10 μg/lane) [44, 45]. The membrane was probed with the following antibodies: anti-FGF2 antibody (#sc-0079, 1:1000; Santa Cruz Biotechnology, Heidelberg, CA, USA), anti-TYMS antibody (ab58287, 1:1000; Abcam, Cambridge, MA, USA), anti-E-cadherin antibody (1:1000; Abcam), anti-N-cadherin antibody (1:1000; Abcam), anti-vimentin antibody (1:1000; Cell Signaling Technology, Beverly, MA, USA), anti-slug antibody (1:1000; Cell Signaling Technology), anti-phosphorylated (p)Smad antibody (1:1000; Abcam), anti-twist antibody (1:1000; Abcam), anti-phospho-p44/42 MAPK (Tyr202/Tyr204) antibody (1:1000; Cell Signaling Technology), anti-p44/42 MAPK antibody (1:1000; Cell Signaling Technology), anti-phospho-Akt antibody (1:1000; Cell Signaling Technology), anti-Akt antibody (1:1000; Cell Signaling Technology), anti-MEK antibody (1:1000; Cell Signaling Technology), anti-phospho-MEK antibody (1:1000; Cell Signaling Technology), anti-phospho-EGFR antibody (Tyr1068) (1:1000; Cell Signaling Technology), and anti-EGFR antibody (1:1000; Cell Signaling Technology), and anti-β-actin (1:5000; Sigma, Saint Louis, MO, USA) was used as a loading control. Each experiment was repeated independently at least three times and one representative blot was selected for the figures.

### Transfection of siRNA

The ON-TARGET plus siRNA for FGF2 (L-006695), TYMS (L-004717), ZEB1 (L-006564), and the negative control (D-001810) were purchased from GE Healthcare (Buckinghamshire, UK). Transfection of each siRNA (5 or 10 nM) was performed using Lipofectamine RNAi-MAX (Thermo Fisher Scientific, Waltham, MA, USA) following the manufacturer’s instructions. At 48 h after transfection, the total RNA or proteins were extracted and 4 × 10^3^ cells/well were cultured in 96-well tissue culture plates and incubated for 72 h after adding variable concentrations of pemetrexed. Finally, the absorbance was measured by using the WST solution, as described above.

### Measurement of FGF2 by ELISA

The concentrations of FGF2 in the conditioned media were measured using an ELISA kit (R&D Systems, Minneapolis, MN, USA). Cells were plated in 24-well plates in the media containing 10% FBS. When the cells reached subconfluence, the media were replaced with RPMI-1640 media without FBS, and then the cells were incubated for a further 24 h. The concentrations of FGF2 in the supernatants were measured using an ELISA kit in accordance with the manufacturer’s protocols.

### Statistical analysis

Data were tested for significance by performing unpaired Student’s *t*-tests; a *p*-value of < 0.05 was considered to indicate statistically significant differences between groups (version 32; SPSS, Chicago, IL, USA).

## SUPPLEMENTARY MATERIALS FIGURES



## Abbreviations

ABCC11: ATP-binding cassette-transporter 11
EMT: epithelial-mesenchymal transition
ERK: extracellular signal-regulated kinase
FGF: fibroblast growth factor
FGFR: fibroblast growth factor
MAPK: mitogen-activated protein kinase
MEK: mitogen-activated protein/extracellular signal–regulated kinase
MTA: pemetrexed
NSCLC: non-small cell lung cancer
PI3K: phosphoinositide 3-kinase
RT-PCR: real-time reverse transcription-polymerase chain reaction
siRNA: small interfering RNA
TKI: tyrosine kinase inhibitor
TS: thymidylate synthase
WST assay: tetrazolium salt-based proliferation assay
ELISA: enzyme-linked immunosorbent assay
