# Supplementary material for: FGF2-FGFR1 pathway activation together with thymidylate synthase upregulation is induced in pemetrexed-resistant lung cancer cells
Source: Oncotarget. 2019 Feb 5;10(11):1171–92. doi: 10.18632/oncotarget.26622 (PMC6383826; doi:10.18632/oncotarget.26622)
Supplement: Supplementary file 1 [file oncotarget-10-1171-s001.pdf]

FGF2-FGFR1 pathway activation together with thymidylate synthase upregulation is induced in pemetrexed-resistant lung cancer cells

SUPPLEMENTARY MATERIALS

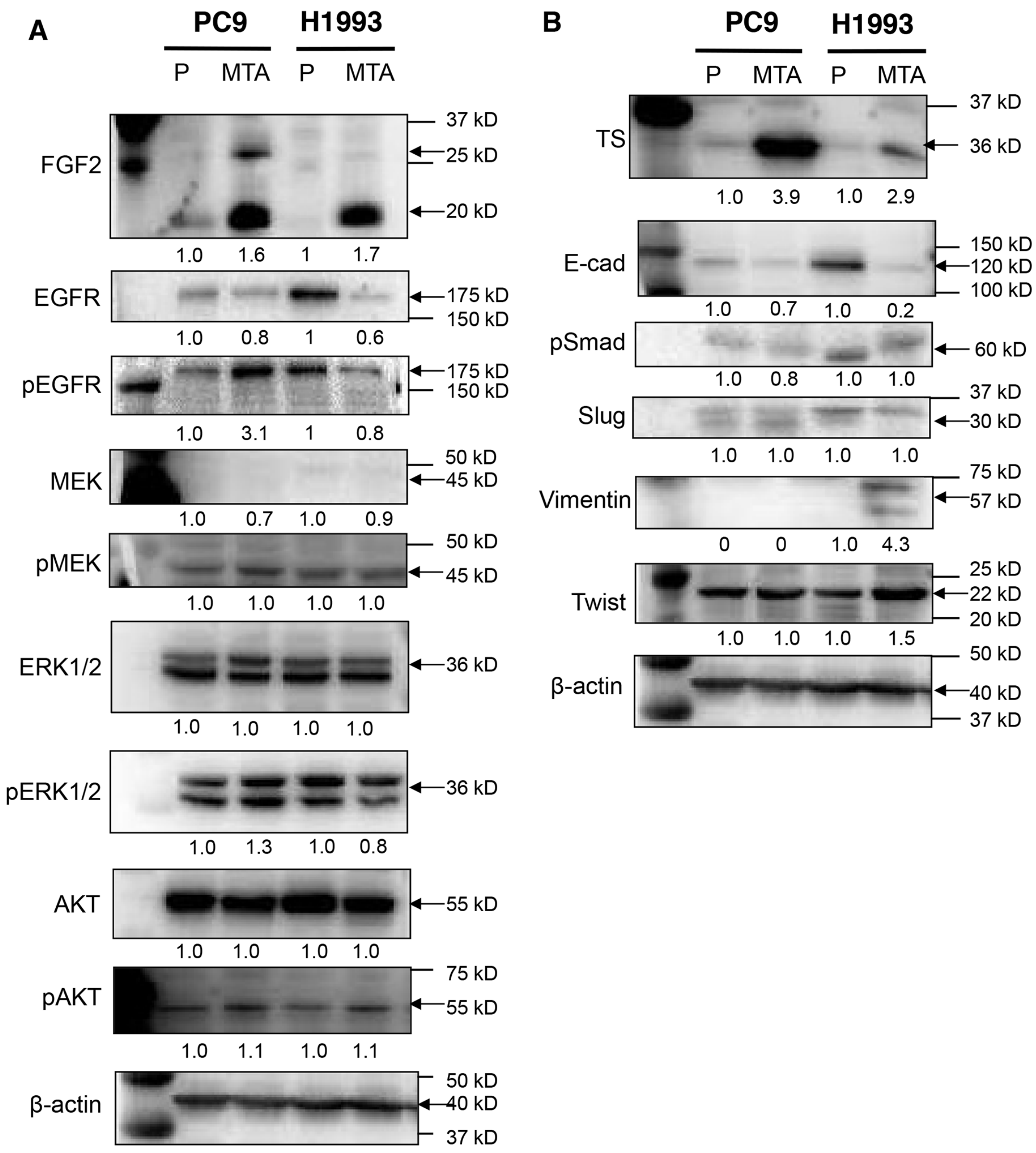

Supplementary Figure 1: Comparison of expression of signaling pathway molecules and EMT marker proteins between parental and pemetrexed-resistant lung cancer cells. (A) Western blots for the expression of total and phosphorylated forms of signaling molecules (pEGFR, pMEK, pERK, and pAKT) in the parental PC9 cells (PC9-P), PC9-MTA cells, parental H1993 cells (H1993-P), and H1993-MTA cells, shown with molecular weight markers. β-actin was used as a loading control. The relative expression levels corrected to β-actin levels are shown below the blots. The phosphorylated protein levels were normalized to their total amounts. Arrows indicate the molecular weight of each protein of interest, and the lines without arrowhead indicate the molecular weight provided by the molecular weight marker (Precision Plus Protein Dual Color Standards, Bio-Rad Laboratories, Inc., Tokyo, Japan). **(B)** Western blots for the expression of TS and EMT marker proteins in PC9-P, PC9-MTA, H1993-P, and H1993-MTA cells, shown with molecular weight.

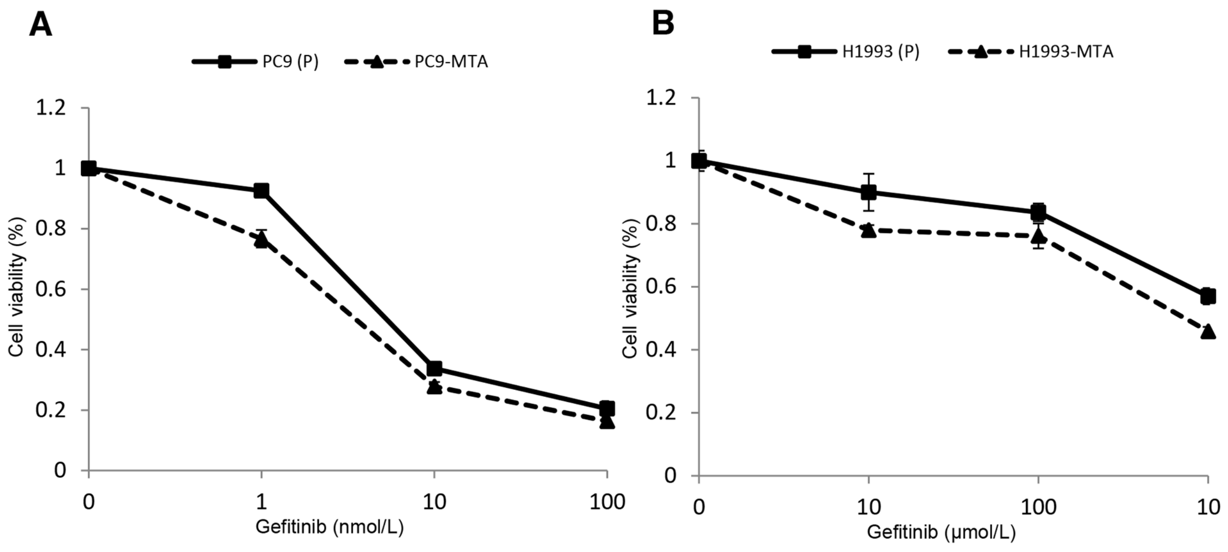

Supplementary Figure 2: Sensitivity to gefitinib in pemetrexed-resistant lung cancer cells. **(A, B)** Sensitivity to gefitinib was tested in the parental PC9 and PC9-MTA cells **(A)** and in the parental H1993 and H1993-MTA cells **(B)** by the WST assay.The error bars represent the standard error of the value obtained in the experiments performed in triplicate.
